# Supplementary material for: Ultralight soft electrostatic actuators based on solid-liquid-gas architectures
Source: Nat Commun. 2026 Feb 19;17:1929. doi: 10.1038/s41467-026-69463-4 (PMC12923549; doi:10.1038/s41467-026-69463-4)
Supplement: Supplementary file 1 — Supplementary Information [file 41467_2026_69463_MOESM1_ESM.pdf]

Supplementary Information for

Ultralight soft electrostatic actuators based on solid-liquid-gas architectures

Hyeong-Joon Joo, Toshihiko Fukushima, Xiying Li, Alona Shagan Shomron, Soo Jin Adrian Koh, Philipp Rothemund*, and Christoph Keplinger*

*To whom correspondence may be addressed:

*Philipp Rothemund
Email: philipp.rothemund@iams.uni-stuttgart.de

*Christoph Keplinger
Email: ck@is.mpg.de

This supplementary information includes:

Supplementary Note

Supplementary Figures 1 to 18

Supplementary Table 1

Supplementary References

Other supplementary materials for this manuscript include the following:

Supplementary Movies 1 to 3

Supplementary Note

Modeling of Peano-HASEL actuator based on solid-liquid-gas architectures

In this Supplementary Note, quasi-static and dynamic models are derived to i) evaluate the effect of compressibility of gaseous dielectric on the quasi-static force-strain characteristic of the model system, ii) evaluate the effect of lower dielectric strength of gaseous dielectric and predict the actuation failures, and iii) evaluate the effect of inertia of liquid dielectric on step response of the model system.

Compressibility of gaseous dielectric

A quasi-static analytical model for a Peano-HASEL actuator is reported to agree well with experimental results[1,2]. We adopt the energy minimization method from the previous model but with an additional *p*-*V* work term to reflect the compressibility of gas.

First, we parameterize an actuator based on the solid-liquid-gas architecture (Supplementary Fig. 2a). An actuator unit is a rectangular pouch with given pouch length L_p and width w (out of the plane of the page), made with solid dielectric shell with thickness of t. The pouch is covered on both sides with electrodes of length L_e and width w. Finally, the pouch is filled with incompressible liquid dielectric of volume V_{liq}, and gaseous dielectric of initial volume $V_{gas.0}$ under the atmospheric pressure ($p_0 = p_{atm}$) and room temperature ($T_0 = T_{room}$). We neglect bending stiffness of the thin solid dielectric shells and assume they are inextensible. We also neglect boundary effects at the edges of the pouch. With these assumptions, the pressure of the liquid or gaseous dielectric deforms the pouch into the two intersecting circular arcs with a central angle of $2\alpha_0$, a radius $r_0 = L_p/2\alpha_0$. With the given geometrical parameters, the total volume inside the pouch V_{total} is calculated to be

$$V_{\mathrm{total}} = \frac{1}{2} w L_{\mathrm{p}}^2 \left(\frac{\alpha_0 - \sin(\alpha_0)\cos(\alpha_0)}{\alpha_0^2} \right) \qquad (1)$$

The total volume of liquid and gas is chosen to form a circular cylinder when the electrodes are fully zipped[3] ($V_{\text{total}} = V_{\text{liq}} + V_{\text{gas.0}} = wL_{\text{p}}{}^2/4\pi$). The initial length of the actuator l_0 when it is fabricated (under the initial pressure $p_0 = p_{\text{atm}}$ and initial temperature $T_0 = T_{\text{room}}$) can be calculated from α_0

$$l_0 = L_{\text{p}}\left(\frac{\sin\alpha_0}{\alpha_0}\right) \tag{2}$$

In some application cases where the actuator actuates under different pressure and temperature conditions other than the fabricated condition ($p_{\text{ext}} \neq p_0$ ($= p_{\text{atm}}$) and/or $T_{\text{ext}} \neq T_0$ ($= T_{\text{room}}$)), the initial length of actuator will be changed as described in Supplementary Fig. 2b, as the volume of the gas inside the actuator will be changed according to the condition (the actuator is at an equilibrium with the surroundings).

$$V_{\text{gas.1}} = V_{\text{gas.0}}\frac{P_0}{P_{\text{ext}}}\frac{T_{\text{ext}}}{T_0} \tag{3}$$

accordingly, the initial central angle α_0 will be changed to α_1 that can be calculated from the following relation

$$V_{\text{gas.1}} = \frac{wL_{\text{p}}{}^2}{2}\left(\frac{\alpha_1 - \sin\alpha_1\cos\alpha_1}{\alpha_1{}^2}\right) - V_{\text{liq}} = V_{\text{gas.0}}\frac{P_0}{P_{\text{ext}}}\frac{T_{\text{ext}}}{T_0} \tag{4}$$

With the calculated α_1 ($\leq \pi/2$), the changed initial length of actuator l_1 can be calculated as

$$l_1 = L_{\text{p}}\left(\frac{\sin\alpha_1}{\alpha_1}\right) \tag{5}$$

When an external load $F = M\text{g}$ and a voltage Φ are applied to the initial state of the actuator, the electrode zips over a length l_{e}. Along with the zipping, the pressure p and the volume of the gas inside the pouch V_{gas} are changed while the volume of the liquid V_{liq} remains the same. The central angle α and the radius r of the arcs change accordingly (Supplementary Fig. 2c).

The corresponding free energy terms of the system then can be described with the given parameters. The total Helmholtz free energy of the system includes mechanical and electrical energy stored in the actuator, free energy of battery, work done on the load, and p-V work against the external pressure, as following equation:

$$U_{\text{total}} = U_{\text{mech}} + U_{\text{elec}} + U_{\text{battery}} + W_{\text{load}} - W_{\text{pV}} \tag{6}$$

Here, the mechanical energy stored in the actuator, U_{mech}, mainly consists of elastic energy stored in the solid dielectric which can be assumed to be negligible in the thin inextensible shell. When charges Q flow from a battery of constant voltage Φ to the actuator, the electric energy stored in the actuator, U_{elec}, can be simplified as energy stored in the zipped region since the electric field outside of the zipped region decays quickly.

$$U_{\text{elec}} = \frac{Q^2}{2C} \tag{7}$$

where the capacitance of the zipped region is

$$C(l_{\text{e}}) = \varepsilon_0 \varepsilon_{\text{s}} \frac{w l_{\text{e}}}{2t} \tag{8}$$

where ε_0 is the permittivity of free space, ε_{s} is the relative dielectric constant of the solid dielectric. This assumption is valid until the unzipped length of the electrodes reduces to the thickness of the film and fringe fields begin to influence the capacitance of the actuator.

The free energy of the battery, U_{battery}, decreases by $Q\Phi$, and with $Q = C\Phi$ relation, it can be described as

$$U_{\text{battery}} = Q\Phi = -C\Phi^2 \tag{9}$$

The work done on the load is

$$W_{\text{load}} = F\left(l_0 - l(\alpha, l_{\text{e}})\right) \tag{10}$$

where $l(\alpha, l_{\text{e}})$ is the final length of the actuator when it is actuated (under the external pressure p_{ext} and temperature T_{ext}).

$$l(\alpha, l_{\text{e}}) = l_{\text{e}} + \left(L_{\text{p}} - l_{\text{e}}\right)\left(\frac{\sin\alpha}{\alpha}\right) \tag{11}$$

The p-V work done against the external pressure p_{ext} is

$$W_{\mathrm{pV}} = (p - p_{\mathrm{ext}})\big(V_{\mathrm{gas}}(\alpha, l_{\mathrm{e}}) - V_{\mathrm{gas.0}}\big) \tag{12}$$

where the $V_{\mathrm{gas}}(\alpha, l_{\mathrm{e}})$ can be described as

$$V_{\mathrm{gas}}(\alpha, l_{\mathrm{e}}) = \frac{w\big(L_{\mathrm{p}} - l_{\mathrm{e}}\big)^2}{2}\left(\frac{\alpha - \sin\alpha\cos\alpha}{\alpha^2}\right) - V_{\mathrm{liq}} \tag{13}$$

With the above equations (7)-(13), the total free energy of the system (equation (6)) can be described with the given parameters and the two independent geometric variables α and l_{e}:

$$\begin{aligned} U_{\mathrm{total}}(\alpha, l_{\mathrm{e}}) = & -\frac{1}{2}\varepsilon_0\varepsilon_{\mathrm{s}}\frac{w l_{\mathrm{e}}}{2t}\Phi^2 + F\left(L_{\mathrm{p}}\left(\frac{\sin\alpha_0}{\alpha_0}\right) - \left(l_{\mathrm{e}} + \big(L_{\mathrm{p}} - l_{\mathrm{e}}\big)\left(\frac{\sin\alpha}{\alpha}\right)\right)\right) \\ & - (p - p_{\mathrm{ext}})\left(\left(\frac{w\big(L_{\mathrm{p}} - l_{\mathrm{e}}\big)^2}{2}\left(\frac{\alpha - \sin\alpha\cos\alpha}{\alpha^2}\right) - V_{\mathrm{liq}}\right) - V_{\mathrm{gas.0}}\right) \end{aligned} \tag{14}$$

Then the equation (14) can be used to find an equilibrium of the system that minimizes the total Helmholtz energy with respect to the two independent geometric variables α and l_{e}

$$\frac{\partial(U_{\mathrm{total}})}{\partial\alpha} = 0 \tag{15}$$

$$\frac{\partial(U_{\mathrm{total}})}{\partial l_{\mathrm{e}}} = 0 \tag{16}$$

As a result, equations (15) and (16) give the relations

$$F = M\mathrm{g} = \frac{\varepsilon_0\varepsilon_{\mathrm{s}} w\Phi^2}{4t}\left(\frac{\cos\alpha}{1 - \cos\alpha}\right) \tag{17}$$

$$p = p_{\mathrm{ext}} + \frac{F}{w(L_{\mathrm{p}} - l_{\mathrm{e}})}\left(\frac{\alpha}{\cos\alpha}\right) \tag{18}$$

with equation (17), the first independent geometric variable α can be calculated as a function of given external load $F = M$g and voltage Φ:

$$\alpha = f(F, \Phi) = \min\left(\frac{\pi}{2}, \cos^{-1}\left(\frac{F}{\frac{\varepsilon_0 \varepsilon_s w \Phi^2}{4t} + F}\right)\right) \tag{19}$$

with equation (18), the other geometric variable l_e can be calculated, either with isothermal or isentropic assumptions.

A. Isothermal actuation

During isothermal actuation, the actuator deforms sufficiently slowly to always be thermally equilibrated to the surroundings (at constant external pressure p_{ext} and temperature T_{ext}).

Assuming ideal gas behavior, the equation of the state of the contained gas can be described as

$$\frac{pV_{gas}}{T} = \frac{p_{gas.0} V_{gas.0}}{T_0} \tag{20}$$

Combining equations (13), (18), and (20) leads to

$$p = p_{ext} + \frac{F}{w(L_p - l_e)}\left(\frac{\alpha}{\cos\alpha}\right) = p_0 \frac{V_{gas.0}}{\frac{w\left(L_p - l_e\right)^2}{2}\left(\frac{\alpha - \sin\alpha\cos\alpha}{\alpha^2}\right) - V_{liq}} \frac{T_{ext}}{T_0} \tag{21}$$

B. Isentropic actuation

During isentropic actuation, the actuator deforms sufficiently fast to prevent any heat exchange with the surroundings. Assuming ideal gas behavior, the equation of the state of the contained gas can be described as

$$pV_{gas}^{\gamma} = p_0 V_{gas.0}^{\gamma} \text{ (where } \gamma = \frac{c_p}{c_v}\text{)} \tag{22}$$

Combining equations (13), (18), and (22) leads to

$$p = p_{ext} + \frac{F}{w(L_p - l_e)}\left(\frac{\alpha}{\cos\alpha}\right) = p_0 \left(\frac{V_{gas.0}}{\frac{w\left(L_p - l_e\right)^2}{2}\left(\frac{\alpha - \sin\alpha\cos\alpha}{\alpha^2}\right) - V_{liq}}\right)^{\gamma} \tag{23}$$

With the calculated $\alpha(F, \Phi)$ from equation (19), equations (21) or (23) can be used to calculate l_e as a function of given external load F, voltage Φ, volume of liquid V_liq, external pressure p_ext, and in case of isothermal actuation, temperature T_ext.

$$l_\mathrm{e} = g\big(F, \Phi, V_\mathrm{liq}, p_\mathrm{ext}, (T_\mathrm{ext})\big) \tag{24}$$

Eventually, equations (19) and (24) uniquely define the two independent geometric variables α and l_e at the given actuation conditions; $(\alpha(F, \Phi),\ l_\mathrm{e}\big(F, \Phi, V_\mathrm{liq}, p_\mathrm{ext}, (T_\mathrm{ext})\big))$.

and with the equation (5), the resulting actuator stroke χ can be calculated

$$\chi = l_1 - l(\alpha, l_\mathrm{e}) = L_\mathrm{p}\left(\frac{\sin\alpha_1}{\alpha_1}\right) - \left(l_\mathrm{e} + \left(L_\mathrm{p} - l_\mathrm{e}\right)\left(\frac{\sin\alpha}{\alpha}\right)\right) \tag{25}$$

where α_1 is calculated with equation (4).

Thus, strain ε is calculated as

$$\varepsilon = \frac{\chi}{l_1} = 1 - \frac{l(\alpha, l_\mathrm{e})}{l_1} = 1 - \frac{l_\mathrm{e} + \left(L_\mathrm{p} - l_\mathrm{e}\right)\left(\frac{\sin\alpha}{\alpha}\right)}{L_\mathrm{p}\left(\frac{\sin\alpha_1}{\alpha_1}\right)} \tag{26}$$

This derived model provides useful insights on how compressibility of gas affects performance of actuator under various thermodynamic conditions, which are shown in Supplementary Figures 3 and 4. With the given materials system and geometry of the actuators described in Methods section in the main text, the change in force-strain characteristic is predicted to be negligible across the entire range of 0% to 100% gas-fill ratios under temperature and pressure conditions that are identical to the conditions where the actuator is fabricated ($p_\mathrm{ext} = p_0$ and $T_\mathrm{ext} = T_0$), for both isothermal and isentropic processes (Supplementary Fig. 3).

Notably, isentropic deformation and isothermal deformation lead to very similar force-strain curves compared to a reference actuator with 0% air-fill. In a real application, the conditions will lie between the isothermal and isentropic extremes, which shows that compressibility is negligible under the analyzed conditions.

In Supplementary Fig. 18, the changes in force-strain characteristic under different external temperature and pressure conditions is predicted using 100% gas-filled actuator as the most extreme case regarding environment effects. Even though the actuator does not work in experiments with 100% gas fill (due to dielectric breakdown through the gas gap), in our model it allows a conservative estimation of the influence of the compressibility of the gas on the actuation performance, when the external pressure and temperature change. Again, materials system and geometry of the actuators are identical to the ones described in Methods section in the main text. Assuming fabrication under standard room conditions (p_0 = 1.0 bar, T_0 = 20 °C) and isothermal actuation under different external pressure and temperature conditions, we examined deviations under design conditions enveloping representative outdoor conditions. We varied the external pressure (p_{ext} = 0.8 to 1.1 bar) at the same external temperature (T_{ext} = 20 °C), and the external temperature (T_{ext} = -30 to 70 °C) at the same external pressure (p_{ext} = 1.0 bar), and compared the resulting force-strain curves.

Supplementary Fig. 18 a and b show that changes in external pressure and temperature change both the maximum generated actuation forces and the maximum actuation strain, as the contained gas changes volume. These variations remain small for typical variations of the environmental conditions. It is possible to counteract changes in actuation behavior through fabrication and design changes, as we show with an example that could occur, in extreme environments such as underwater. As shown in the Supplementary Fig. 18c, increasing the external pressure to 3.0 bar at an external temperature of 20 °C considerably changes the force-strain curve of an actuator that was filled with gas p_0 = 1.0 bar and T_0 = 20 °C. The resulting decrease of the gas volume increases the blocking force, but drastically reduces the maximum actuation stroke, because the electrodes are fully zipped before the angle α reaches 90 °. By increasing the electrode length, the full range of actuation strains can be achieved (dashed line in Supplementary Fig. 18c). Additionally, when a high external pressure is anticipated, the actuator can be pre-filled at a higher pressure to match the external pressure (p_{fill} = 3.0 bar to match the p_{ext} = 3.0 bar); compressibility effects then become negligible, and the original force-strain behavior is recovered.

Lower dielectric strength of gaseous dielectric

Dielectric breakdown of gaseous dielectric is governed by Paschen's law[4]; Paschen's law provides breakdown voltage Φ_B as a function of gas pressure and electrode gap. In our model system the potential difference across the gas (air) Φ_{air} at the liquid-gas interface (where breakdown is most probable) is calculated to be

$$\Phi_{air} = \frac{d\varepsilon_s}{2t\varepsilon_{air} + d\varepsilon_s}\Phi \qquad (27)$$

where ε_s and ε_{air} are the dielectric constants of the solid dielectric and air, respectively, d is the distance between solid dielectric shells along the liquid-gas interface, t is the thickness of the solid dielectric shell, Φ is the voltage applied to the system (Supplementary Fig. 7a). To find the conditions where Φ_{air} reaches Φ_B at the liquid-gas interface, we need to specify the p and d at a given actuation condition (F, Φ, V_{liq}), so that we can find the intersection between $\Phi_{air}(\Phi, d)$ (equation (27)) and $\Phi_B(p, d)$ given by Paschen's law. Since we can calculate p as a function of (F, Φ, V_{liq}) from the model derived in the previous section (equation (18)), we only need to derive d as a function of (F, Φ, V_{liq}). Using the shape of the actuator at a given condition (equations (19) and (24)), d can be calculated with a geometrical analysis (Supplementary Fig. 7b). With given $\alpha = f(F, \Phi)$ (equation (19)) and $l_e = g(F, \Phi, V_{liq})$ (equation (24)), y in Supplementary Fig. 7b can be derived as a function of x, so that we can calculate x_{int} for a known V_{liq} with the following relation

$$V_{liq} = \int_0^{x_{int}} 2y(x) \cdot w \, \mathrm{d}x \qquad (28)$$

and then d is derived

$$d = 2 \cdot y(x_{int}) \qquad (29)$$

where x and y are distances from the zipping front along the x-, and y-axis, respectively, x_{int} is the location of the liquid-gas interface from the zipping front along the x-axis. For simplicity in calculation, the following relations were derived from the geometry:

$$r(\alpha, l_{\mathrm{e}}) = \frac{L_{\mathrm{p}} - l_{\mathrm{e}}}{2\alpha} \tag{30}$$

$$\theta(x) = \sin^{-1}\left(\sin\alpha - \frac{x}{r}\right) \tag{31}$$

$$y(\theta) = r(\cos\theta - \cos\alpha) \tag{32}$$

With the derived relations (equations (30)-(32)), the equation (28) becomes

$$V_{\mathrm{liq}} = \int_{\alpha}^{\theta_{\mathrm{int}}=\theta(x_{\mathrm{int}})} 2y(\theta)\cdot w\,(-r\cos\theta\ \mathrm{d}\theta)$$

$$= wr^2\{(\alpha - \theta_{\mathrm{int}}) + \sin\theta_{\mathrm{int}}\,(2\cos\alpha - \cos\theta_{\mathrm{int}}) - \sin\alpha\cos\alpha\} \tag{33}$$

and the equation (29) becomes

$$d = 2\cdot y(\theta_{\mathrm{int}}) \tag{34}$$

We merged the above variables with equations (30)-(33) and solved it using the ODE45 solver in MATLAB R2020b (MathWorks, Inc.). It gave us θ_{int} for a known V_{liq}, which could be used for calculating d with equations (32) and (34).

As a result, we could calculate p and d of actuators at given conditions, i.e., $p(F, \Phi, V_{\mathrm{liq}})$, $d(F, \Phi, V_{\mathrm{liq}})$, and map the values into the Paschen's plane to predict if the actuator would undergo a dielectric breakdown through gas or not.

Inertia of liquid dielectric

We observed that the peak strain rate of the actuators differs with different air-fill ratios. We hypothesize that the difference comes from the differences in inertia of liquid dielectric rather than differences in viscosity, since all the actuators fall within the inertia domain defined in previous work[5]. To understand the effect of inertia of liquid dielectric we developed a simplified dynamic model that includes the mass of liquid dielectric

m_{liq} (Supplementary Fig. 11a). The motion of the system can be calculated using the Lagrangian equation of the second kind.

$$\frac{\mathrm{d}}{\mathrm{d}t}\left(\frac{\partial L}{\partial \dot{\chi}}\right) - \frac{\partial L}{\partial \chi} = q \tag{35}$$

With $L = T - P$, where T and P are the kinetic and potential energy of the system, respectively,

$$L = T - P = \frac{1}{2}M\dot{\chi}^2 + \frac{1}{2}m_{\mathrm{liq}}\dot{h}^2 - \Pi_{\mathrm{total}} \tag{36}$$

where M and m_{liq} are masses of the deadweight and liquid dielectric, respectively, χ and h are traveled distances of the deadweight and liquid dielectric, respectively (Supplementary Fig. 11a), Π_{total} is total potential energy of the system which is a sum of potential energy of liquid dielectric $m_{\mathrm{liq}}\mathrm{g}h$ and potential energy of the other components from the previous section (equation (14)), and q represents viscous dissipation force which we consider negligible in this model for simplicity.

Now, each separate term from the Lagrangian equation of motion (equation (35)) can be calculated as:

$$\frac{\mathrm{d}}{\mathrm{d}t}\left(\frac{\partial L}{\partial \dot{\chi}}\right) = \quad M\ddot{\chi} + m_{\mathrm{liq}}\frac{\mathrm{d}}{\mathrm{d}t}\left(\dot{h}\frac{\partial \dot{h}}{\partial \dot{\chi}}\right) \tag{37}$$

$$\frac{\partial L}{\partial \chi} = \quad -\frac{\partial \Pi_{\mathrm{total}}}{\partial \chi} = \frac{\varepsilon_0\varepsilon_{\mathrm{s}}w\Phi^2}{4t}\frac{\partial l_{\mathrm{e}}}{\partial \chi} - F + \left(p_0\frac{V_{\mathrm{gas.0}}}{V_{\mathrm{gas}}} - p_{\mathrm{ext}}\right)\frac{\partial V_{\mathrm{gas}}}{\partial \chi} - m_{\mathrm{liq}}\mathrm{g}\frac{\partial h}{\partial \chi} \tag{38}$$

where all the variables are described in the previous sections. For simplicity, we neglect the compressibility of gaseous dielectric, as we showed the compressibility has limited effect on quasi-static force-strain performance of the actuators. Thus, the values that were described as functions of both α and l_{e} are now considered to be functions of only α, while $\frac{\partial V_{\mathrm{gas}}}{\partial \chi}$ can be neglected. With the geometrical relations $\chi = L_{\mathrm{p}}\left(\frac{\sin\alpha_0 - \alpha_0}{\alpha_0}\right) - l_{\mathrm{p}}\left(\frac{\sin\alpha - \alpha}{\alpha}\right)$ and $h = (L_{\mathrm{p}} - l_{\mathrm{p}}) + \chi$ where $l_{\mathrm{p}} = L_{\mathrm{p}}\frac{\alpha}{\alpha_0}\left(\frac{\alpha_0 - \sin\alpha_0\cos\alpha_0}{\alpha - \sin\alpha\cos\alpha}\right)^{1/2}$, each separate term from equations (37) and (38) can be denoted as followings, as functions that are only depending on α:

$$\ddot{\chi} = L_{\mathrm{p}}\left(\frac{\alpha_0-\sin\alpha_0\cos\alpha_0}{\alpha-\sin\alpha\cos\alpha}\right)^{1/2}\left\{\frac{(\sin\alpha-\alpha\cos\alpha)(1-\cos\alpha)}{\alpha-\sin\alpha\cos\alpha}\frac{\ddot{\alpha}}{\alpha_0} + \frac{\sin\alpha\{(\alpha-2\alpha\cos\alpha+\sin\alpha)(\alpha-\sin\alpha\cos\alpha)+3\sin\alpha(\alpha\cos\alpha-\sin\alpha)(1-\cos\alpha)\}}{(\alpha-\sin\alpha\cos\alpha)^2}\frac{\dot{\alpha}^2}{\alpha_0}\right\} \tag{39}$$

$$\frac{\mathrm{d}}{\mathrm{d}t}\left(\dot{h}\frac{\partial\dot{h}}{\partial\dot{\chi}}\right) = L_{\mathrm{p}}\left(\frac{\alpha_0-\sin\alpha_0\cos\alpha_0}{\alpha-\sin\alpha\cos\alpha}\right)^{1/2}\left\{\frac{\sin\alpha-\alpha\cos\alpha}{(\alpha-\sin\alpha\cos\alpha)(1-\cos\alpha)}\frac{\ddot{\alpha}}{\alpha_0} + \frac{\sin\alpha\{(\alpha-\sin\alpha\cos\alpha)(\alpha-\sin\alpha)+3\sin\alpha(\alpha\cos\alpha-\sin\alpha)(1-\cos\alpha)\}}{(\alpha-\sin\alpha\cos\alpha)^2(1-\cos\alpha)^2}\frac{\dot{\alpha}^2}{\alpha_0}\right\} \tag{40}$$

$$\frac{\partial l_{\mathrm{e}}}{\partial\chi} = \frac{\cos\alpha}{1-\cos\alpha} \tag{41}$$

$$\frac{\partial h}{\partial\chi} = \frac{1}{1-\cos\alpha} \tag{42}$$

Substituting the terms from equations (39)-(42) into equations (37) and (38), the Lagrangian equation of motion of the system (equation (35)) becomes

$$C_1\frac{\ddot{\alpha}}{\alpha_0} + C_2\frac{\dot{\alpha}^2}{\alpha_0} + C_3 = q \tag{43}$$

with

$$C_1(\alpha) = U_1U_2\left(M + m_{\mathrm{liq}}\frac{1}{(1-\cos\alpha)^2}\right) \tag{44}$$

$$C_2(\alpha) = U_1U_3\left(M + m_{\mathrm{liq}}\frac{1}{(1-\cos\alpha)^2}\frac{(\alpha-\sin\alpha\cos\alpha)(\alpha-\sin\alpha)+3\sin\alpha(\alpha\cos\alpha-\sin\alpha)(1-\cos\alpha)}{(\alpha-\sin\alpha\cos\alpha)(\alpha-2\alpha\cos\alpha+\sin\alpha)+3\sin\alpha(\alpha\cos\alpha-\sin\alpha)(1-\cos\alpha)}\right) \tag{45}$$

$$C_3(\alpha) = F + \frac{m_{\mathrm{liq}}\mathrm{g}}{1-\cos\alpha} - \frac{\varepsilon_0\varepsilon_{\mathrm{s}}w\Phi^2}{4t}\frac{\cos\alpha}{1-\cos\alpha} \tag{46}$$

where

$$U_1(\alpha) = L_{\mathrm{p}}\left(\frac{\alpha_0-\sin\alpha_0\cos\alpha_0}{\alpha-\sin\alpha\cos\alpha}\right)^{1/2} \tag{47}$$

$$U_2(\alpha) = \frac{(\sin\alpha - \alpha\cos\alpha)(1-\cos\alpha)}{\alpha - \sin\alpha\cos\alpha} \tag{48}$$

$$U_3(\alpha) = \frac{\sin\alpha\{(\alpha - 2\alpha\cos\alpha + \sin\alpha)(\alpha - \sin\alpha\cos\alpha) + 3\sin\alpha(\alpha\cos\alpha - \sin\alpha)(1-\cos\alpha)\}}{(\alpha - \sin\alpha\cos\alpha)^2} \tag{49}$$

With the above, we can now check how m_{liq} affects the Lagrangian equation of motion of the system. Especially, the $\frac{\ddot{\alpha}}{\alpha_0}$ term in equation (43), which represents the acceleration of the system, can provide a key insight into the accelerating phase of actuation. The coefficient of the $\frac{\ddot{\alpha}}{\alpha_0}$ term shown in equation (44), shows that a term of the form $\frac{m_{\mathrm{liq}}}{(1-\cos\alpha)^2}$ is added to the mass M; this term can be considered as an added inertial mass on top of the mass of the external load, and can be described as

$$\frac{m_{\mathrm{liq}}}{(1-\cos\alpha)^2} = m_{\mathrm{add}} = c_1(\alpha) m_{\mathrm{liq}} \tag{50}$$

where $c_1(\alpha) = \frac{1}{(1-\cos\alpha)^2}$

To gain some qualitative insights on the effect of m_{liq}, instead of trying to solve the full equation of motion, we define the modified weight $M_{\mathrm{mod}} = M + m_{\mathrm{add}} = M\left(1 + c_1 \frac{m_{\mathrm{liq}}}{M}\right)$;

Since m_{add} is a function of α, we take the average of this value for a qualitative estimation.

$$\overline{m_{\mathrm{add}}} = \bar{c}_1 m_{\mathrm{liq}} = \frac{m_{\mathrm{liq}}}{\alpha_{\mathrm{f}} - \alpha_0} \int_{\alpha_0}^{\alpha_{\mathrm{f}}} \frac{\mathrm{d}\alpha}{(1-\cos\alpha)^2} \tag{51}$$

Supplementary Table 1 shows the coefficient $\overline{c_1}$ of the additional mass under different external loads. Using the values from the table, the peak strain rate with respect to the modified weight $\overline{M_{\mathrm{mod}}} \cdot \mathrm{g} = (M + \overline{m_{\mathrm{add}}}) \cdot \mathrm{g}$ converges into a single trend line (Supplementary Fig. 11b), showing that the simplified dynamic analysis presented above is sufficient to provide key qualitative insights into the actuation process, and to identify the inertia of the liquid dielectric as the main cause for the observed differences in response speed of air filled actuators.

Evaluation of dynamic performance in the worst orientation

In this section we extend the evaluation of dynamic performance to different orientations, specifically focusing on the worst-case scenario. Here, the "worst orientation" refers to the orientation where the electrode is positioned at the top of the actuator, causing gravity to pull the liquid dielectric away from the zipping front. This configuration allows us to gain insights for optimizing the liquid-gas ratio under various potential use cases. The evaluation procedure is identical with the one described in the *Dynamic performance* section of the main text.

Step response

As expected, the resulting peak strain rate, peak power, and peak specific power exhibited different trends (Supplementary Fig. 15a) from those observed in the best orientation (Fig. 4c-e). For clarity, we maintain the same categorization: we identify a liquid-dominant group (0%-60% air-filled actuators) and a gas-dominant group (70%-90% air-filled actuators). In the worst orientation, the peak strain rate of the liquid-dominant group reached levels similar to those of the gas-dominant group in the best orientation. This trend aligns with the discussion from the Supplementary Note of *Inertia of liquid dielectric*; in the worst orientation, the inertia of liquid no longer opposes actuation, enabling the liquid-dominant group to perform comparably to the gas-dominant group in the best orientation, where inertia of liquid is small. Conversely, the gas-dominant group in the worst orientation showed slower peak strain rates, likely due to dielectric breakdown within the gaseous dielectric. Peak power also followed this trend, with the liquid-dominant group achieving the performance of the gas-dominant group in the best orientation, while the gas-dominant group underperformed. However, the underperformance of the gas-dominant group with respect to absolute power numbers is compensated by their lighter weight, resulting in peak specific power values comparable to those of the liquid-dominant group; we note a lower reliability of achieved actuation strain, as indicated by larger error bars.

Frequency response

The normalized amplitude results also exhibited different trends (Supplementary Fig. 15b) from the best orientation (Fig. 4g). The normalized amplitude for actuators with 20%, 40%, 60%, and 70% air-fill is somewhat lower than the 0% air-filled sample, likely due to dielectric breakdown of the gaseous dielectric; still, all samples with air-fills up to 70% featured useful actuation response up to 60Hz. In contrast, the 80% and 90% air-filled actuators showed a rapid decline in normalized amplitude.

Prolonged actuation at constant voltage and in cyclic actuation

Prolonged actuation at constant voltage

To test force relaxation related to charge accumulation[6], we measured the actuation stroke of actuators under constant voltage and force for 600 seconds. We compared an actuator with 0% air-fill (reference) with actuators with 70% air-fill in the best and worst orientations. At $t = 0$, a constant voltage of 8 kV and a constant force of 1 N were applied, using the high voltage amplifier and the dual-mode lever system described in the Methods section in the main text (also see Supplementary Fig. 1). For all three cases, the normalized amplitude—normalized with the initial stroke of each actuator—remained nearly constant over the duration of 600 seconds. After 600 seconds, the amplitudes of the 0% air-filled actuator (reference) and the 70% air-filled actuator in the best orientation dropped by 3.3% and 0.0004% from their initial amplitudes, respectively, while the 70% air-filled actuator in the worst orientation showed an increase of 1.6%. These findings suggest that the introduction of air has a negligible influence on the charge accumulation behavior.

Prolonged actuation in cyclic actuation

To assess long-term durability of the actuators, we performed 100,000-cycle-long durability tests with actuators with 0% air (reference) and 70% air in the best and worst orientations (7 samples for each fill and orientation). The fabrication procedure was identical to those described for the frequency response experiments specified in Methods section in the main text. A sinusoidal voltage signal (6 kV peak, single polarity) at 5 Hz was used for actuation (Supplementary Fig. 6b). A nearly constant force of 1 N was applied using the springs

specified in Supplementary Fig. 12. The spring resonance frequency was well separated from the 5 Hz actuation frequency.

Under this operating condition, all seven tested samples for each condition—0% air, 70% air in best and worst orientations—remained functional after 100,000 cycles. For each condition, stroke measurements were obtained from one representative sample, using the laser displacement sensor described in the Methods section. The stroke amplitudes, normalized by their respective initial values, remained stable up to 10,000 cycles, after which a gradual decline was observed, likely due to charge retention effects[6]. The level of degradation was similar for all cases, including the reference actuator with 0% air, indicating that the presence of air has negligible impact on long-term durability under these actuation conditions.

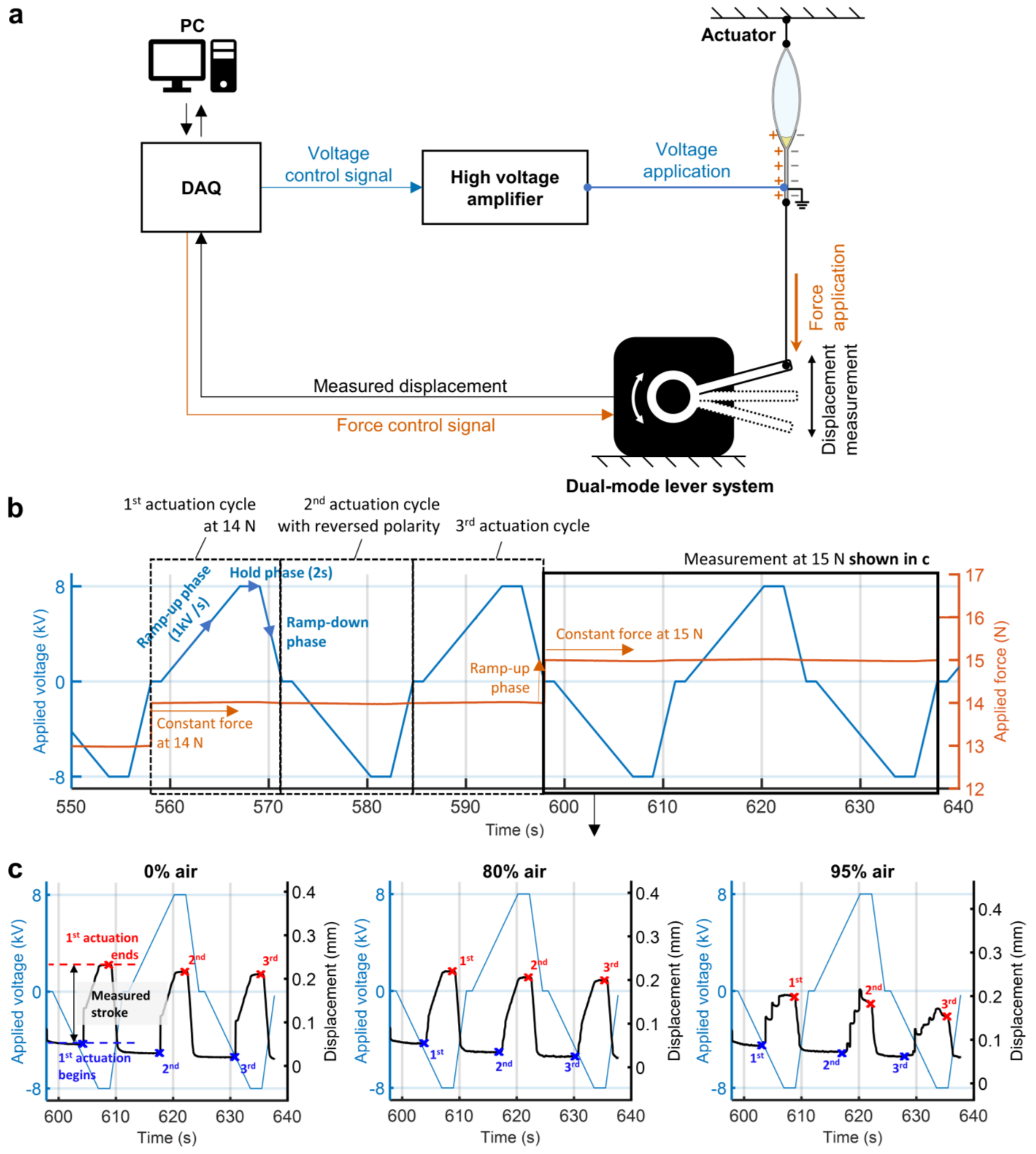

Supplementary Figure 1. Measurement of quasi-static strains.

a Schematic of setup showing voltage and force application, and strain measurement. **b** Input signals: Voltage ramped to 8 kV at 1 kV s^{-1}, held for 2 s, then ramped down to 0 kV, with polarity reversed each cycle to prevent charge accumulation. Three actuation cycles were performed per applied force, which was

incrementally increased by steps of 1 N. **c** Measured displacements at 15 N, with stroke defined as the difference in displacement from the start of actuation (initial rapid change in displacement during the voltage ramp) to the end of actuation (2 s after maximum voltage is reached). Comparison of normal (0% and 80% air) and failed actuation (95% air) is shown. Source data are provided as a Source Data file.

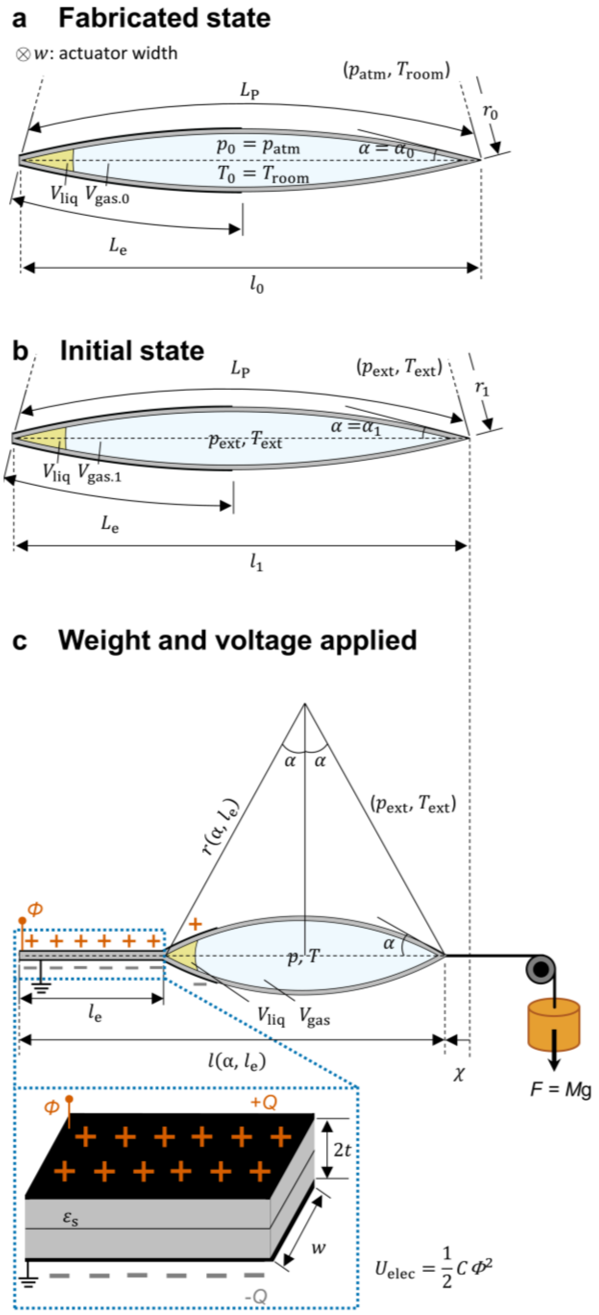

Supplementary Figure 2. Analytical model of three-phase Peano-HASEL actuators.

a Schematic of the actuator at fabricated state, with a given geometry (length L_p, width w, electrode length L_e) and selection of dielectrics (liquid volume V_{liq}, initial gas volume $V_{gas.0}$) under the atmospheric pressure ($p_0 = p_{atm}$) and room temperature ($T_0 = T_{room}$). The angle α parameterizes the shape of the actuator. **b** Schematic of the actuator at initial state, under different pressure and temperature conditions (p_{ext} and T_{ext}). The angle α parameterizes the shape of the actuator. The volume of the gas inside the actuator will be affected by the external pressure and temperature conditions, thus changing the initial central angle α_0 and initial length of

actuator l_0. **c** Actuator during actuation (applied load *F*, voltage *Φ*), with electrodes zipped to a length l_e. The actuation process changes the gas pressure *p*, and thus the volume of gas V_{gas}. Stored electrical energy is determined by the shell material (thickness *t*; dielectric constant ε_s).

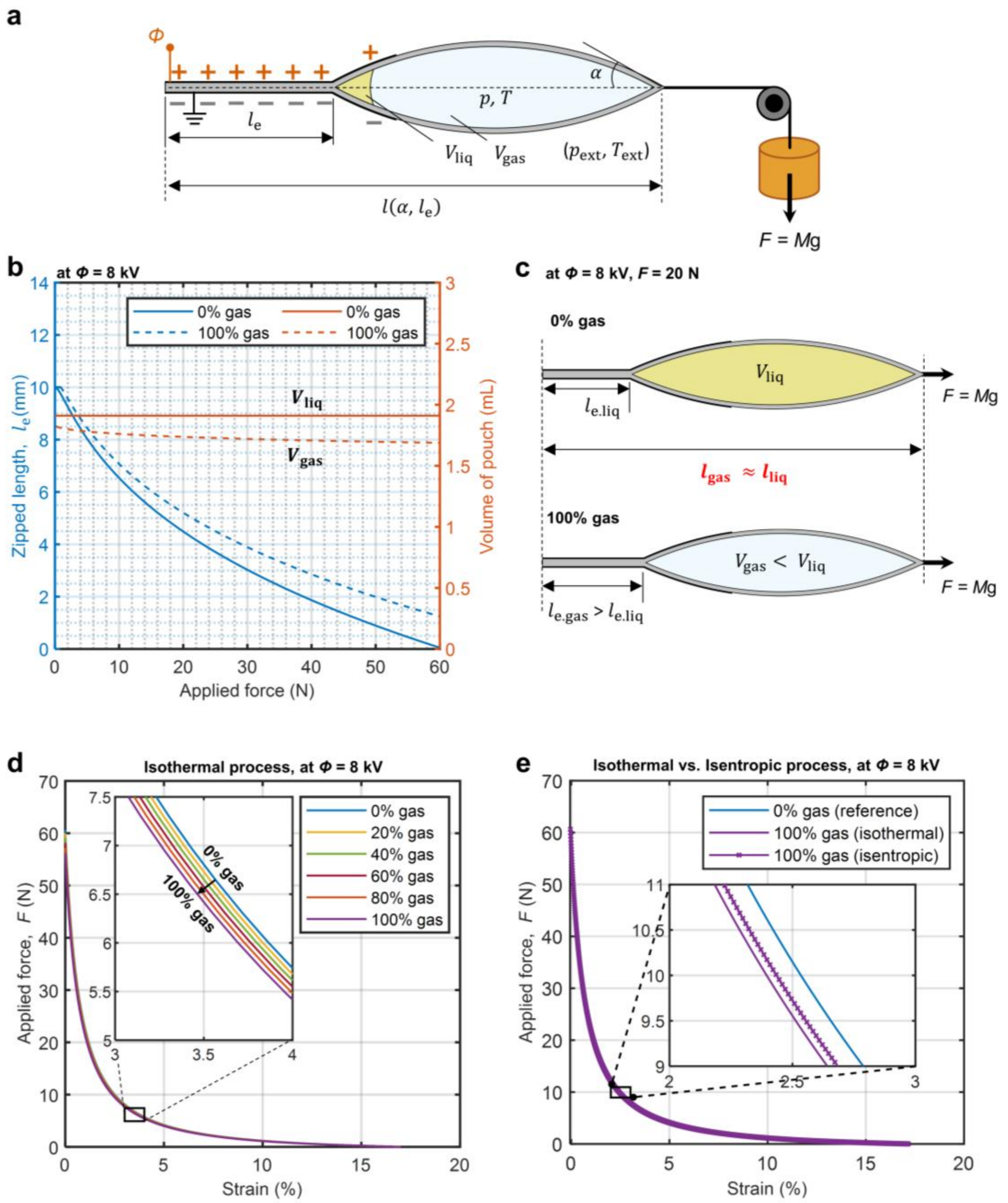

Supplementary Figure 3. Results of the analytical model of three-phase Peano-HASEL actuators.

a Schematic illustration and parameterization of the model. **b** Theoretically calculated volume of pouch and zipped length of the actuators, at 0% (fully liquid-filled) and 100% (fully gas-filled) gas-fill ratios, under 8 kV voltage. The compressibility of the gas leads to pressure and volume changes of the gas during actuation. **c** Pouch shape of actuators at 0% and 100% gas-fill at 8 kV voltage and 20 N force, drawn based on calculations. Despite an increased gas pressure and a resulting reduction in gas volume, the larger zipped length ($l_{e.gas} > l_{e.liq}$) compensates for the compressed gas volume, yielding nearly identical actuator lengths ($l_{gas} \approx l_{liq}$). **d** Force-strain

curves of actuators with varying gas-fills, based on the analytical model, assuming isothermal deformation. **e** Force-strain curves of 100% gas-filled actuators are compared under both isothermal and isentropic processes. Source data are provided as a Source Data file.

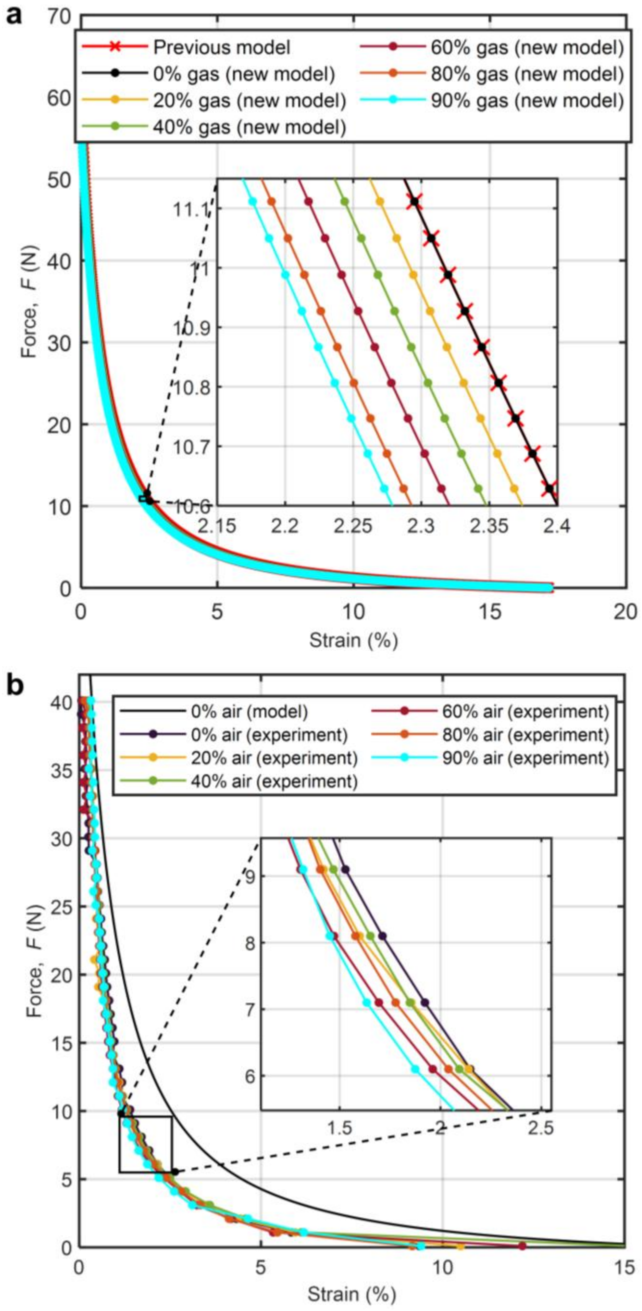

Supplementary Figure 4. Model verification.

a Force-strain curves predicted by the new model incorporating compressibility of gaseous dielectric. The new model converges to the established model for Peano-HASEL actuators[1] (red line) when the amount of gas-fill approaches 0% (black line). **b** Force-strain curves obtained from experiments. As predicted by the model, negligible deviation is observed across gas-fill ratios. Source data are provided as a Source Data file.

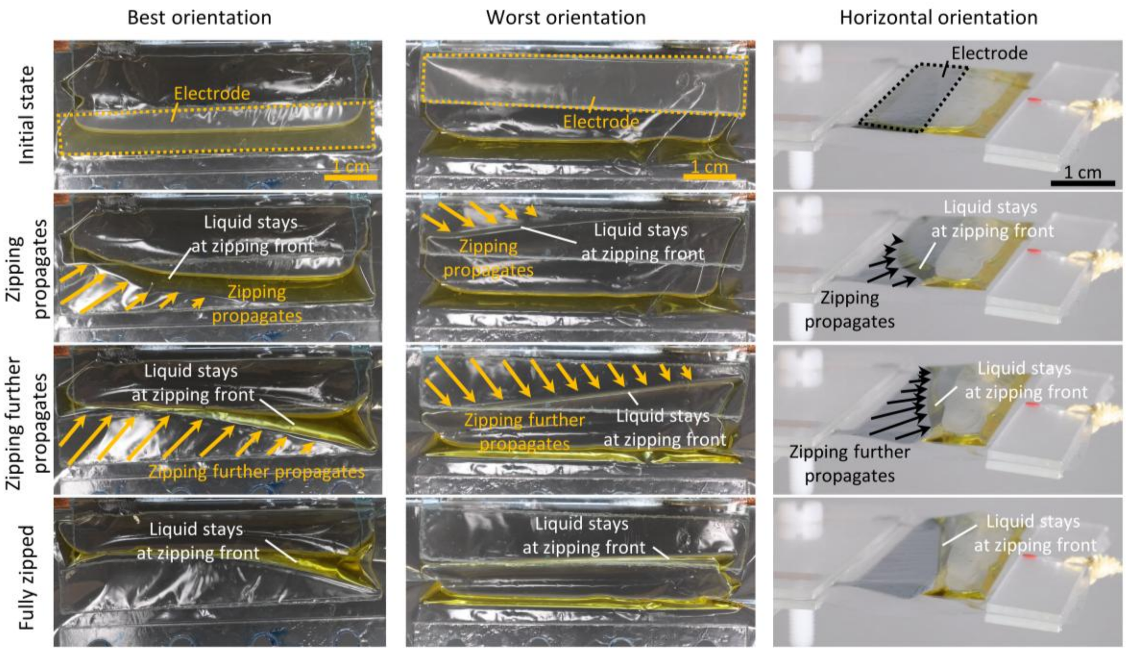

Supplementary Figure 5. Retention of liquid dielectric at zipping front.

Video snapshots demonstrating the presence of liquid at the zipping front throughout actuation, in the best, worst, and horizontal orientation. Best and worst orientation experiments were performed at an earlier stage of research, using hydrogel electrodes; horizontal orientation experiments were performed later using pedot:PSS electrodes—these different experimental conditions should, however, not influence the presence of liquid at the zipping front.

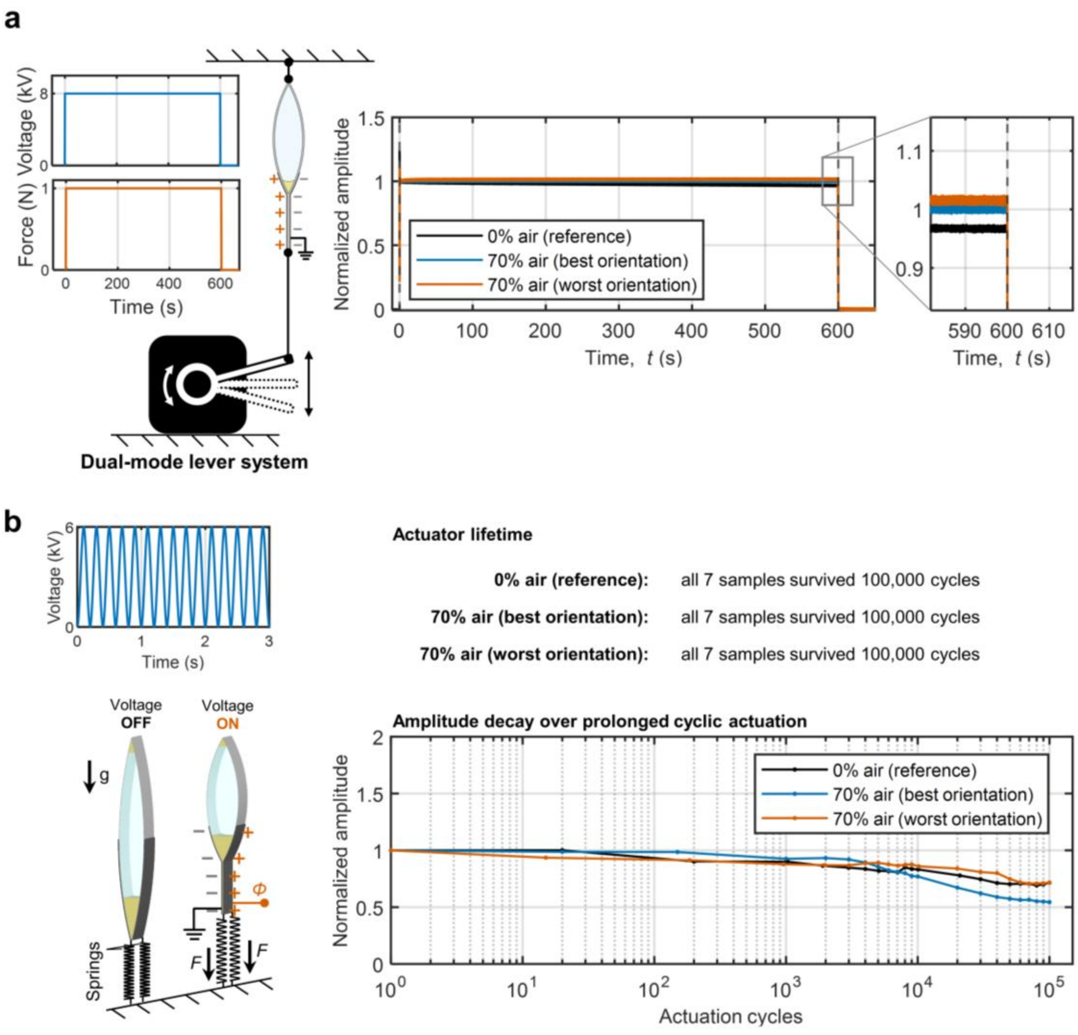

Supplementary Figure 6. Prolonged actuation at constant voltage and in cyclic actuation.

a Actuation strokes normalized by their initial strokes as a function of time, under constant voltage and force. For all three cases, the normalized amplitude remained almost constant, indicating no negative influence from the presence of air. **b** Normalized amplitude over 100,000 cycles under a sinusoidal voltage signal, for actuators with 0% air-fill (reference) and with 70% air-fill. At this specific actuation condition, all seven samples for each case remained functional after 100,000 cycles. Actuator stroke was normalized with the initial stroke of each actuator, to show the actuator behavior over many cycles. For all three cases, the normalized amplitude remained stable up to 10,000 cycles, after which a gradual decline was observed, likely due to charge retention effects[6]. The level of degradation was similar for all cases, including the reference actuator with 0% air, suggesting that the presence of air has negligible impact on long-term durability. Source data are provided as a Source Data file.

a

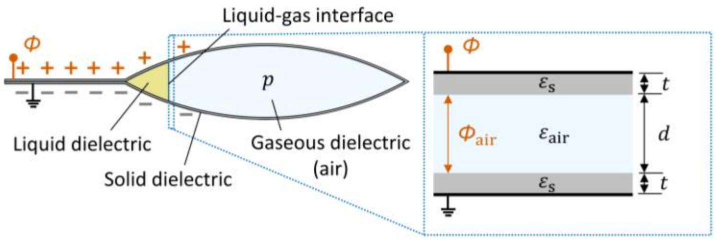

b

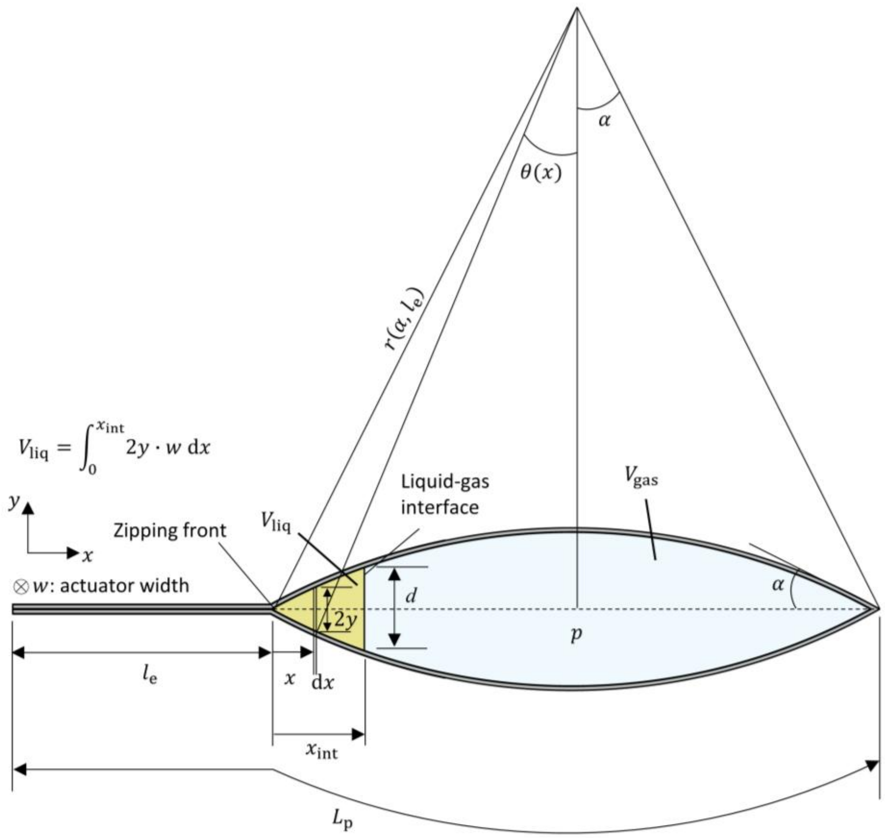

Supplementary Figure 7. Region where the highest electric field occurs in gaseous dielectrics.

a Schematic illustration of the liquid-gas interface, where the highest electric field occurs in gaseous dielectrics (air). The interface is modeled as layers of parallel dielectric plates, with an air layer (dielectric constant of ε_{air}) of thickness d, between the layers of solid dielectric (dielectric constant of ε_s) with thickness t. **b** Schematic of the actuator used to calculate the gap d between the solid dielectric shells at the liquid-gas interface.

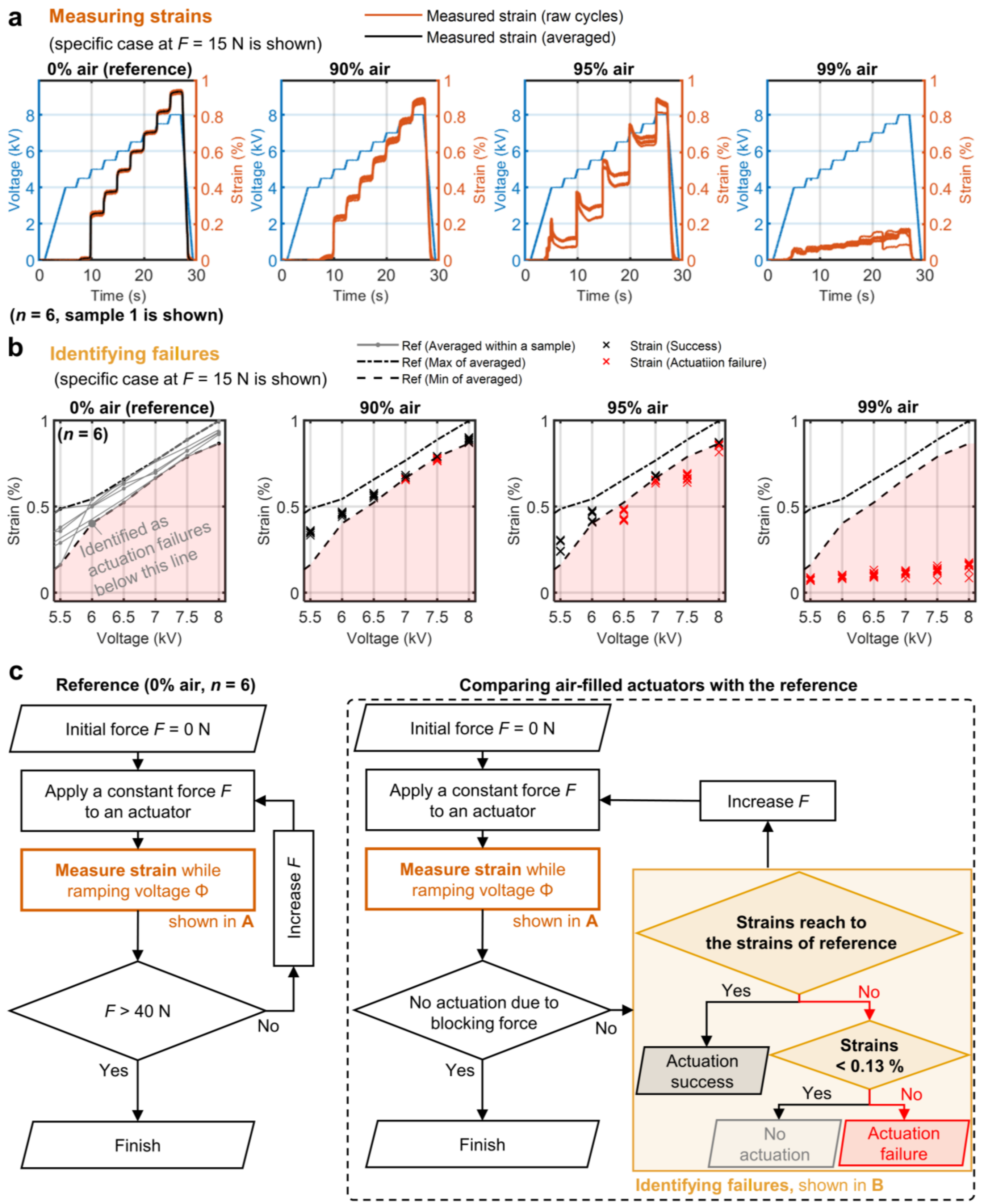
a
Measuring strains
(specific case at F = 15 N is shown)
Measured strain (raw cycles)
Measured strain (averaged)
0% air (reference)
90% air
95% air
99% air
Voltage (kV)
Strain (%)
Time (s)
(n = 6, sample 1 is shown)
b
Identifying failures
(specific case at F = 15 N is shown)
Ref (Averaged within a sample)
Ref (Max of averaged)
Ref (Min of averaged)
Strain (Success)
Strain (Actuatiion failure)
(n = 6)
Identified as actuation failures below this line
Voltage (kV)
c
Reference (0% air, n = 6)
Initial force F = 0 N
Apply a constant force F to an actuator
Measure strain while ramping voltage Φ
shown in A
Increase F
F > 40 N
No
Yes
Finish
Comparing air-filled actuators with the reference
Initial force F = 0 N
Apply a constant force F to an actuator
Measure strain while ramping voltage Φ
shown in A
Increase F
No actuation due to blocking force
No
Yes
Finish
Strains reach to the strains of reference
Yes
No
Actuation success
Strains < 0.13 %
Yes
No
No actuation
Actuation failure
Identifying failures, shown in B

Supplementary Figure 8. Identifying actuation failures.

a Actuation strains of reference actuators (0% air-fill) and actuators with different air-fills (90%, 95%, and 99%) measured at 0.5 kV intervals from 4 kV to 8 kV, and intervals of 1 N from 0 N to 40 N. Strain data at a constant force of 15 N is shown as an example. Six samples were used for measuring reference strains, with ten cycles per sample, with the first two initializing cycles excluded from analysis. **b** Measured strains and identified actuation failures at 15 N are shown as an example. The strains are expected to be larger than the minimum strain observed from the reference; actuation failures were defined when the strain was smaller than the minimum reference strain at the same voltage and force. **c** Flow charts summarizing the process of measuring reference strains and identification of actuation failures. Source data are provided as a Source Data file.

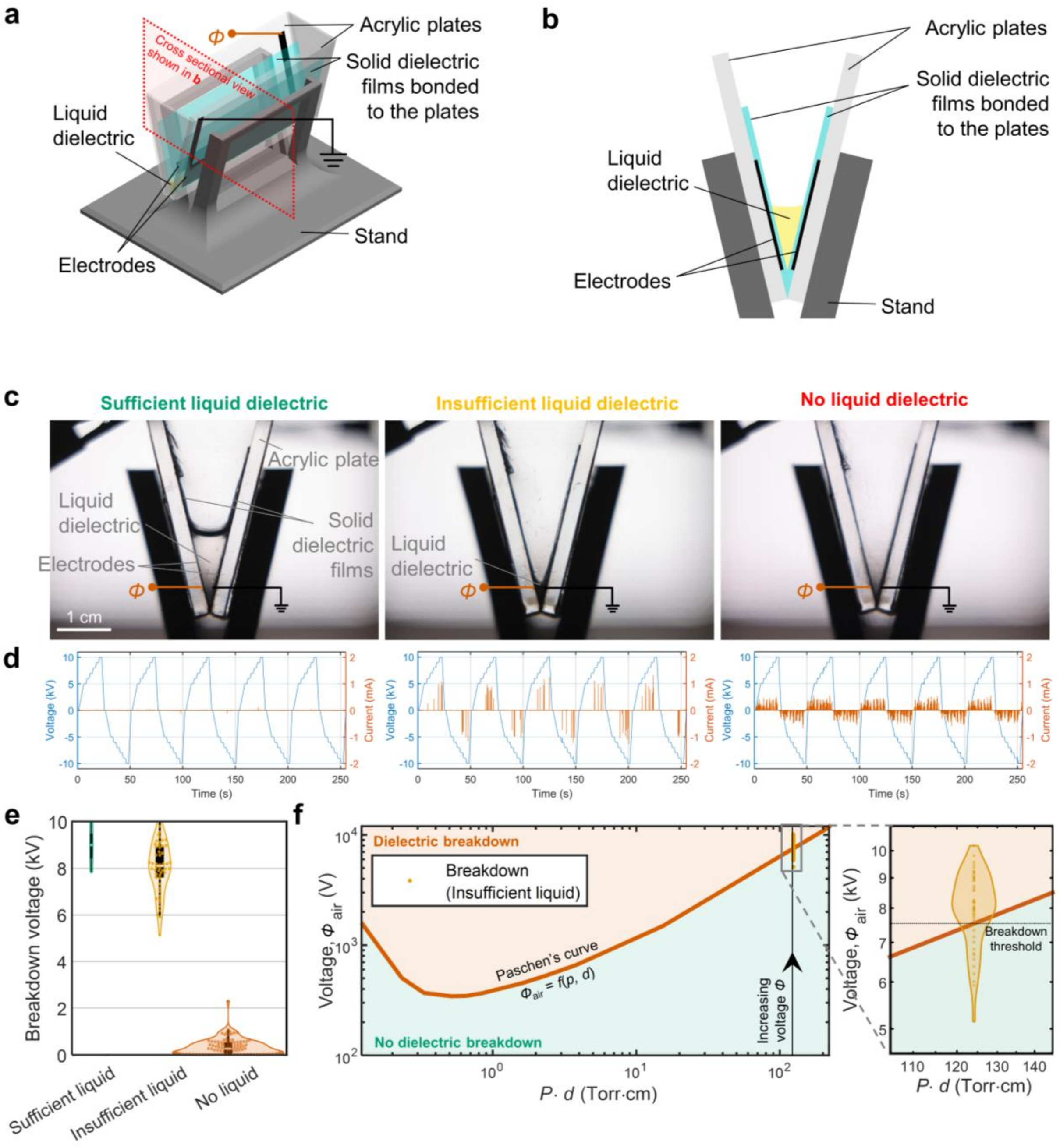

Supplementary Figure 9. Dielectric breakdown test of liquid-gas interfaces in a simplified setup.

A simplified experimental setup using rigid acrylic plates (**a**, **b**) to replicate the zipping front of actuators under three conditions (**c**): sufficient (left), insufficient (middle), and no (right) liquid dielectric. Carbon electrodes were printed on the outer surfaces of solid dielectric films which were securely bonded to the acrylic plates. **d** Measured current profiles for each condition, when actuators were exposed to the typical activation voltage pattern used for actuator evaluation. Therefore, an identical voltage signal ramping at 1 kV s^{-1} and pausing for 2 seconds at every 1 kV step from 5 to 10 kV was applied for 100 cycles (10 voltage cycles are shown in the

figure; however, current data for all 100 cycles is shown, so that all current spikes are visible), with polarity reversed each cycle. The setup with sufficient liquid dielectric (left; electrodes fully covered) exhibited only a few and minor current spikes, indicating effective insulation. In contrast, the setup with insufficient liquid dielectric (middle; electrodes partially covered) exhibited frequent current spikes, particularly at higher voltages. The setup with no liquid dielectric (right) exhibited numerous current spikes, even at low voltages. **e** Voltages at which the first current spikes occurred in each cycle. The white line denotes the median value, and the thick black bars denote the standard deviation; the thin black bar shows the data range from lowest to highest value, avoiding outliers. The width of the colored areas indicates the number of recoded breakdown events for a given voltage. **f** Paschen's curve for air (orange) intersecting with a black line denoting the measured value of atmospheric pressure p, which was measured to be 973.3 hPa (730.0 Torr), and the electrode gap along the liquid-air interface d, which was 1.70 mm. The intersection point of the orange and black lines indicates the predicted impending dielectric breakdown of air within the setup. The zoomed-in view shows that most breakdown events align well with the value predicted from Paschen's law. Source data are provided as a Source Data file.

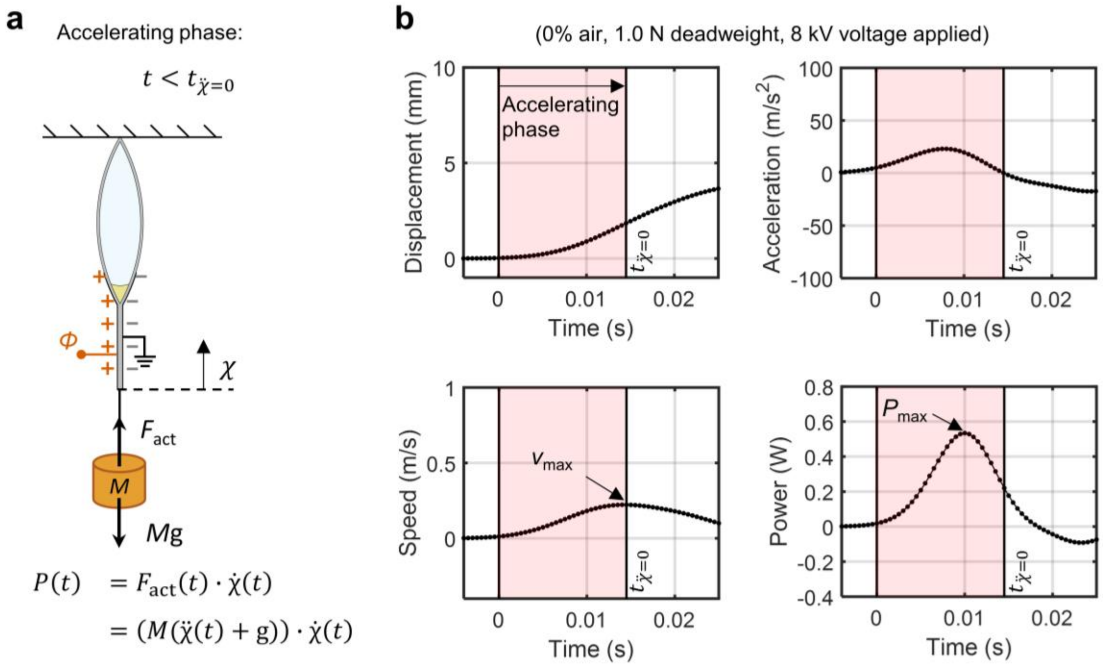

Supplementary Figure 10. Definition of accelerating phase, peak strain rate, and peak power.

a Schematic illustrating the accelerating phase. We can calculate the power $P(t)$ exerted by the actuator to the deadweight, with the given equation, during the defined accelerating phase. **b** The measured displacement, speed, acceleration, and power with respect to time. Displacement was filtered (Savitsky-Golay) and differentiated to obtain speed and acceleration, with power calculated based on speed and acceleration data. Peak values were identified within the accelerating phase. Source data are provided as a Source Data file.

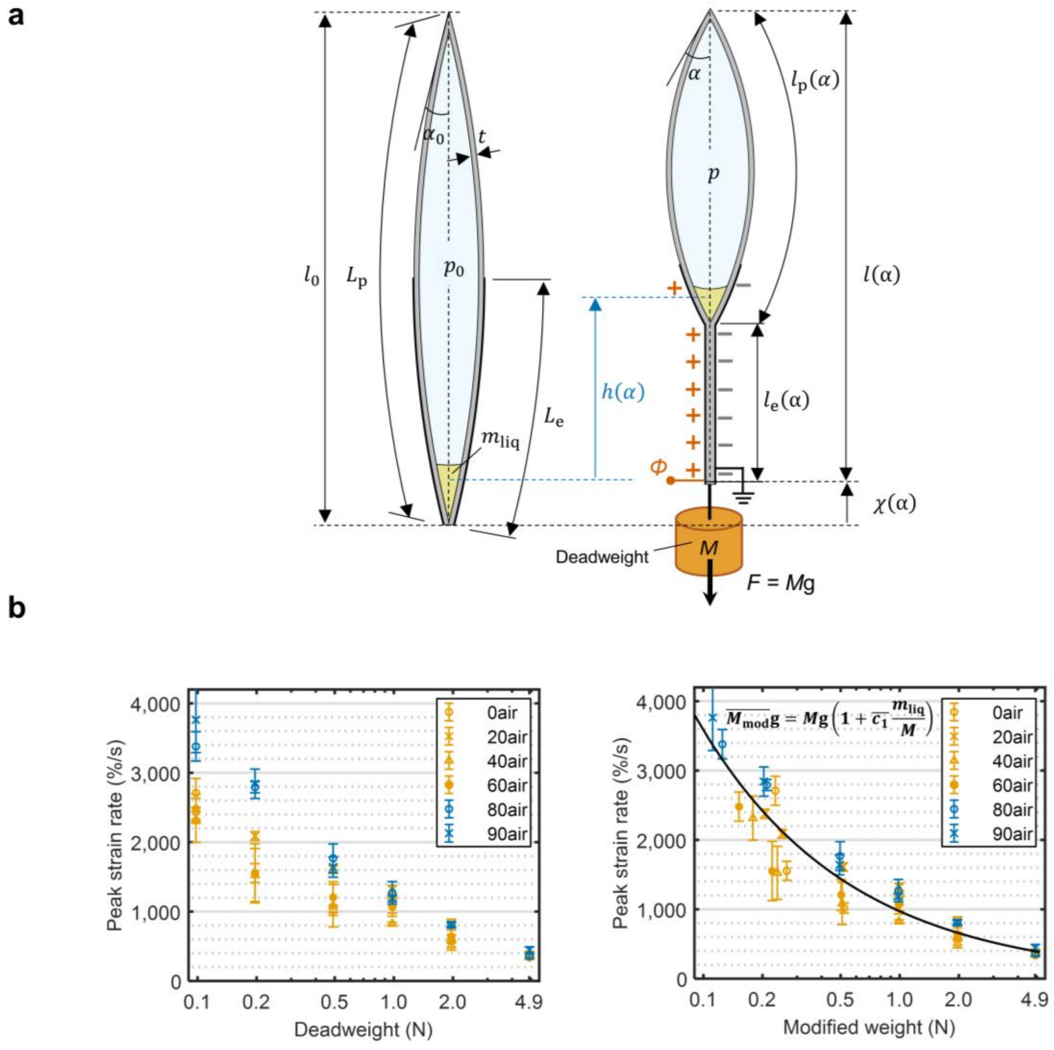

Supplementary Figure 11. Effect of reduced inertia of liquid on actuation performance.

a Schematic of an actuator used for analyzing the effect of inertia of liquid. The liquid mass m_{liq} is raised vertically by $h(\alpha)$ as the electrode zips by a distance of $l_e(\alpha)$. This analysis provides the modified weight, accounting for both the inertia of the liquid and the deadweight. Here, compressibility of air is neglected. **b** Peak strain rates as a function of both original deadweight (left) and modified weight (right). With the modified weight incorporating inertia of the liquid mass, the trend of peak strain rate collapses onto a single line. Error bars represent the standard deviations for $n \geq 5$ experimental trials. Source data are provided as a Source Data file.

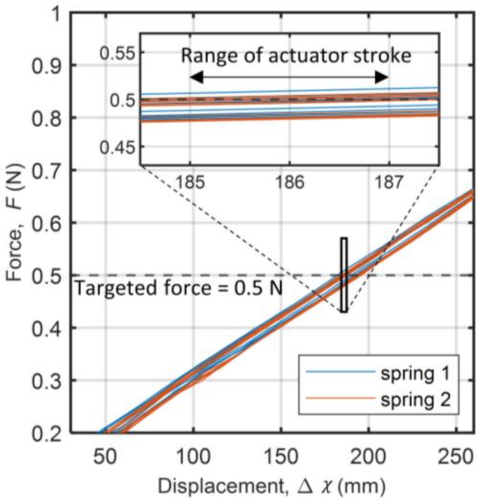

Supplementary Figure 12. Evaluation of springs.

Force-displacement curves of the springs used in frequency response experiments. Two springs were used to apply an approximately constant force of 1 N (0.5 N each) throughout the fast actuation process at high actuation frequencies. We note that complex resonance behavior of such springs could potentially affect the obtained results on frequency response of the actuators. Five actuation cycles are shown to demonstrate the linearity and repeatability of the springs, covering the targeted force region of 0.5 N, across the maximum possible range of actuator stroke. Source data are provided as a Source Data file.

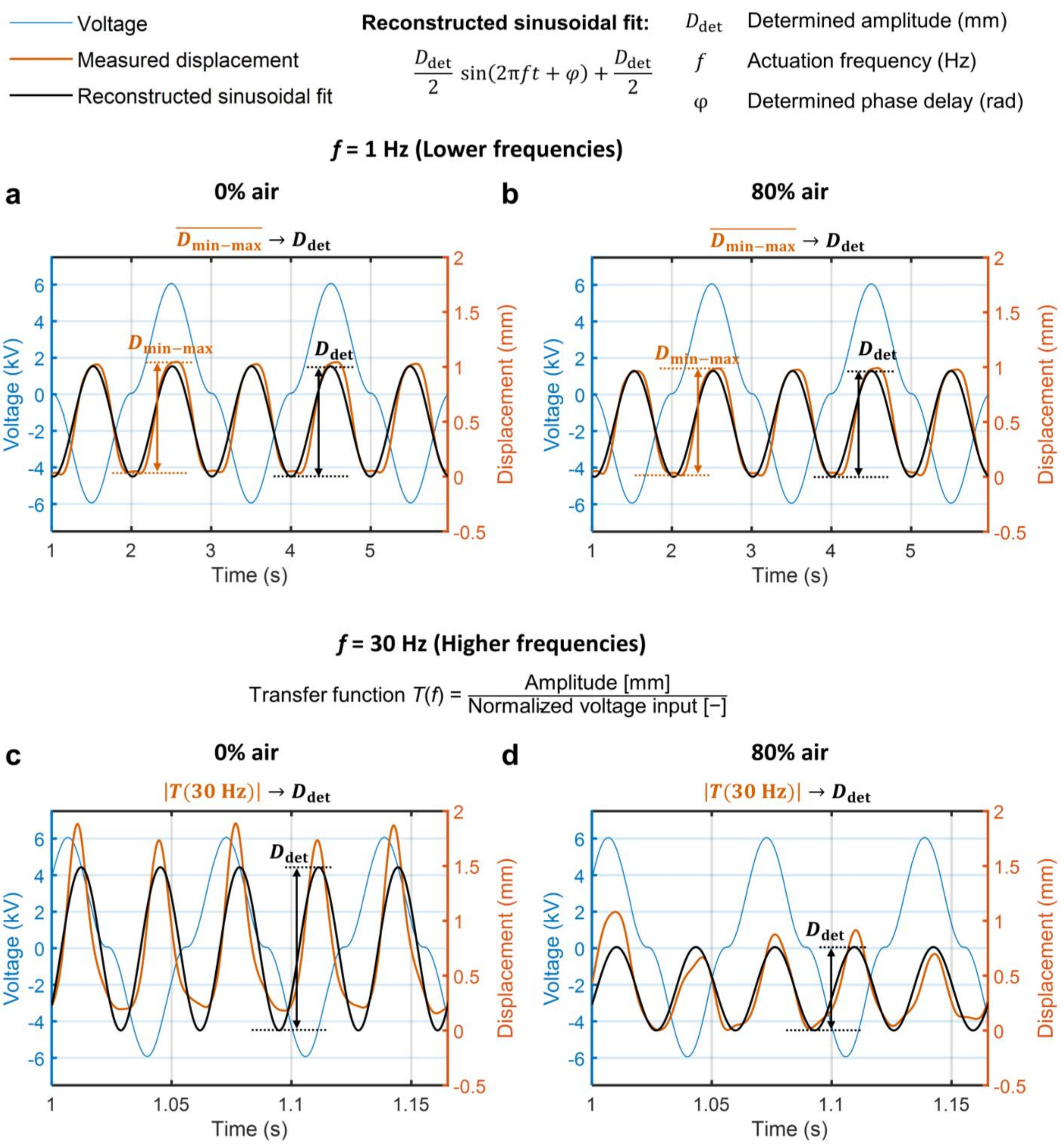

Supplementary Figure 13. Identifying actuation amplitude in frequency response.

a-b Measured displacement signals and strategy to extract amplitude information at lower frequencies (1 Hz). At low frequencies, actuators return to their initial position, resulting in a non-sinusoidal response due to their inextensible nature. Therefore, the determined actuation amplitude D_{det} was measured from the averaged min-max displacement of the actuator ($\overline{D_{min-max}}$). With this strategy, at lower frequencies including 1 Hz, the

reconstructed sinusoidal fits aligned well with the measured displacement signals for both lower (here exemplified with 0% air-fill) and higher (here exemplified with 80% air-fill) air-fills. The 0% and 80% air-filled actuators exhibited similar responses at 1 Hz. **c-d** Measured displacement signals and strategy to extract amplitude information at higher frequencies (30 Hz). At high frequencies, the actuator does not have enough time to return to its initial position, resulting in a more sinusoidal response. Therefore, we used a transfer function identified with the "tfestimate" function in MATLAB (input: normalized voltage, output: amplitude) to identify the actuation amplitude. Despite inconsistent responses from cycle to cycle at 30 Hz, the transfer function reasonably captures the averaged response for both **c** 0% and **d** 80% air-filled actuators. Source data are provided as a Source Data file.

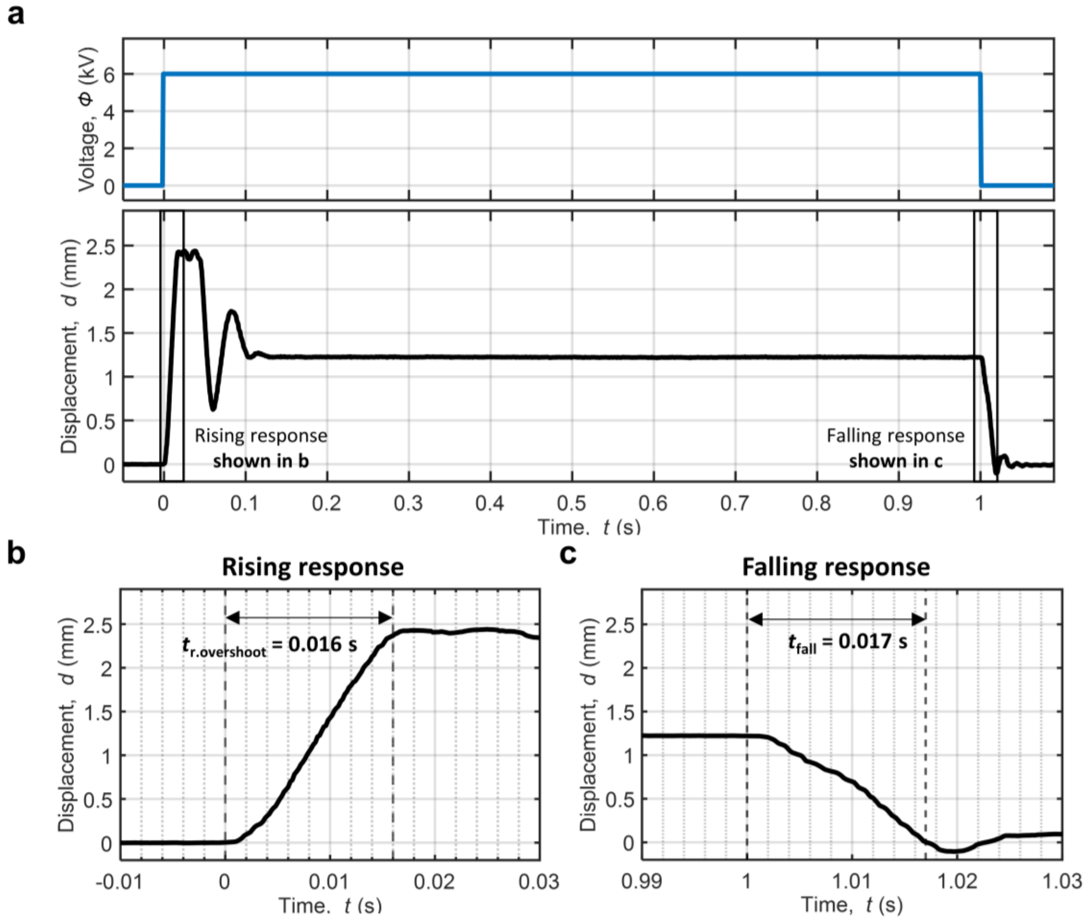

Supplementary Figure 14. Evaluation of rising and falling responses of the actuators when driven with square wave voltage signals.

a Step voltage input (6 kV) lasting for 1 s and the corresponding actuator displacement, with the rising and falling responses shown in **b** and **c**, respectively. **b** Rising response of the actuator. The time to reach the overshoot displacement is $t_{r.overshoot}$ = 0.016 s. **c** Falling response of the actuator when the voltage is turned off. The time to return to the initial position is $t_{falling}$ = 0.017 s. These rising and falling times correspond to half the period of 30 Hz (T/2 = 0.0167 s). Source data are provided as a Source Data file.

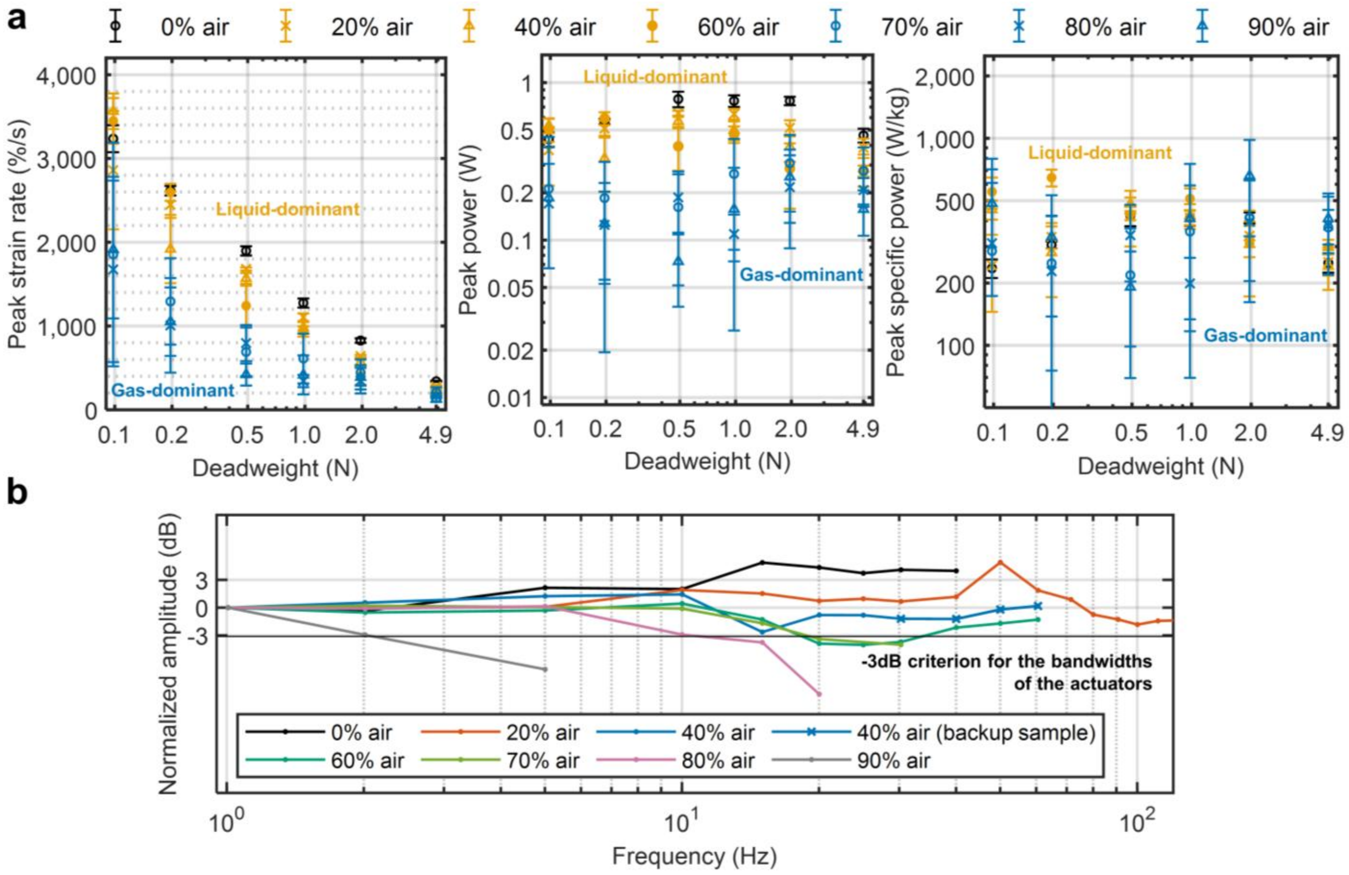

Supplementary Figure 15. Evaluation of dynamic response in the worst orientation.

a Peak strain rate, peak power, and peak specific power from the step response in the worst orientation. Unlike in the best orientation, the gas-dominant actuator group exhibited worse performance compared to the liquid-dominant actuator group. **b** Normalized amplitude in frequency response. The actuator sample with 40% air-fill broke during the test, thus a backup sample was used afterwards. Error bars represent the standard deviations for $n \geq 5$ experimental trials. Source data are provided as a Source Data file.

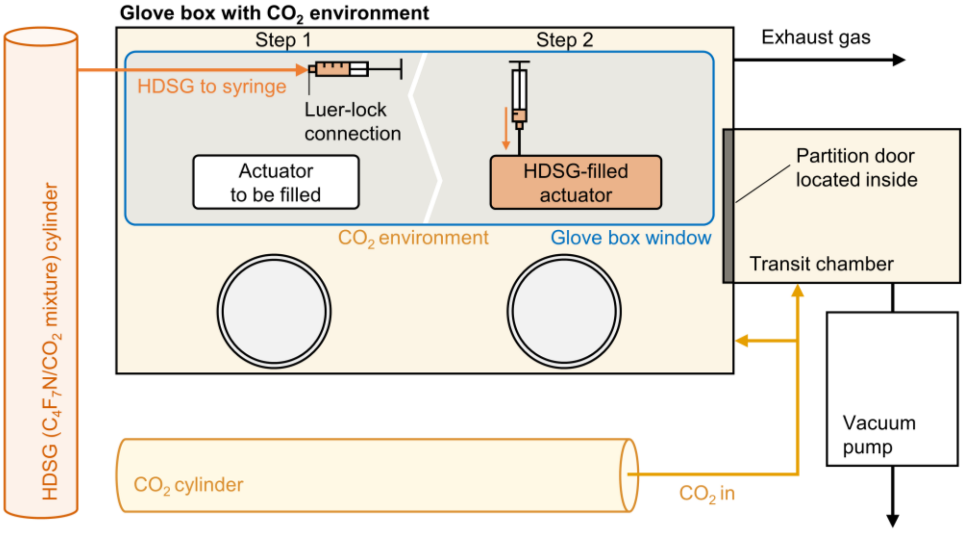

Supplementary Figure 16. Glove box setup for fabrication of HDSG-filled actuators.

Schematic of the glove box setup used to fill actuators with HDSG, a gas mixture of C_4F_7N and CO_2. The glove box is maintained at 1 bar CO_2. A dedicated gas line delivers HDSG into syringes equipped with Luer-lock connectors inside the glove box. Given the 80% CO_2 content of the HDSG, a CO_2 environment in the glove box helps prevent contamination with ambient air.

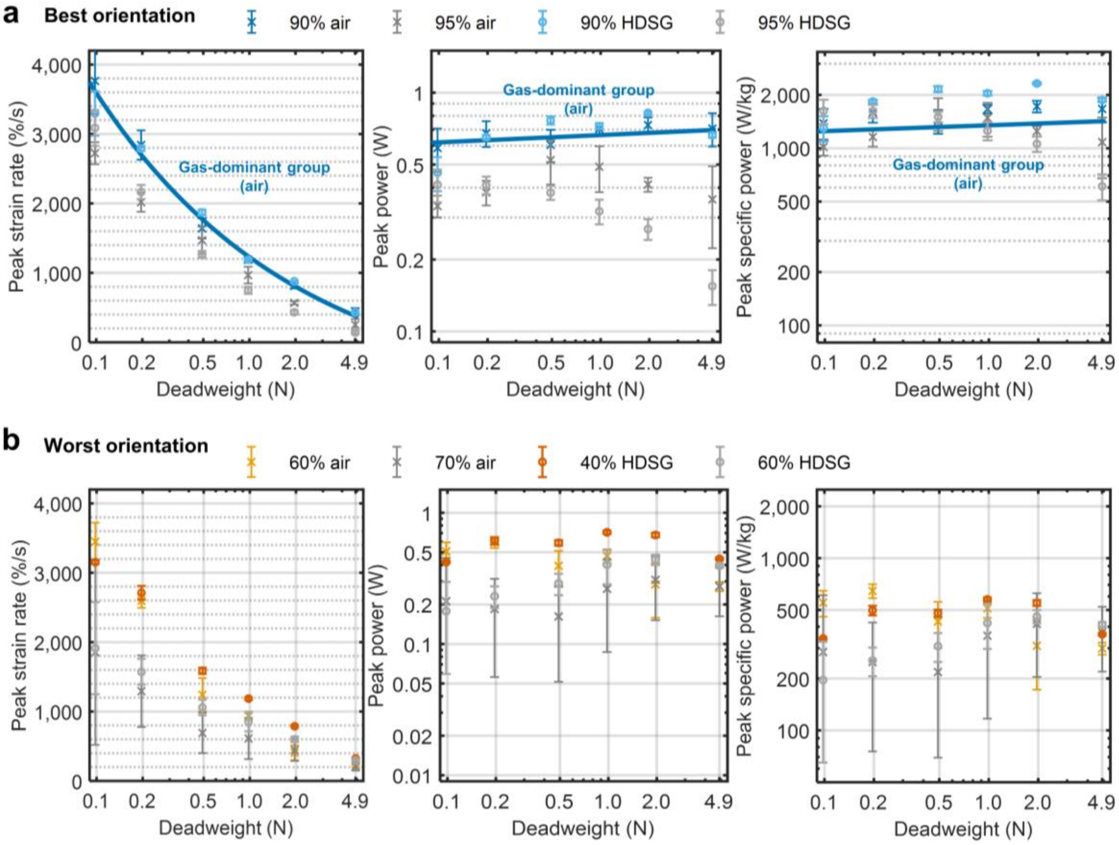

Supplementary Figure 17. Evaluation of actuator performance under step voltage input with high-dielectric-strength gas (HDSG).

Peak strain rate, peak power, and peak specific power of actuators filled with air (marked with crosses) and high-dielectric-strength gas (HDSG; marked with circles), when actuators are driven by a step voltage input. **a** In the best orientation, air-filled actuators exhibited stable performance up to 90% air-fill, following the blue trend line obtained from the gas-dominant group (filled with air) in the main text; actuation failed beyond 90% fill. The use of HDSG did not extend this threshold. **b** In the worst orientation, HDSG-filled actuator showed stable actuation up to 40% fill, which was not improved compared to the 60% of air-filled actuators. Error bars represent standard deviations for $n \geq 5$ trials. Source data are provided as a Source Data file.

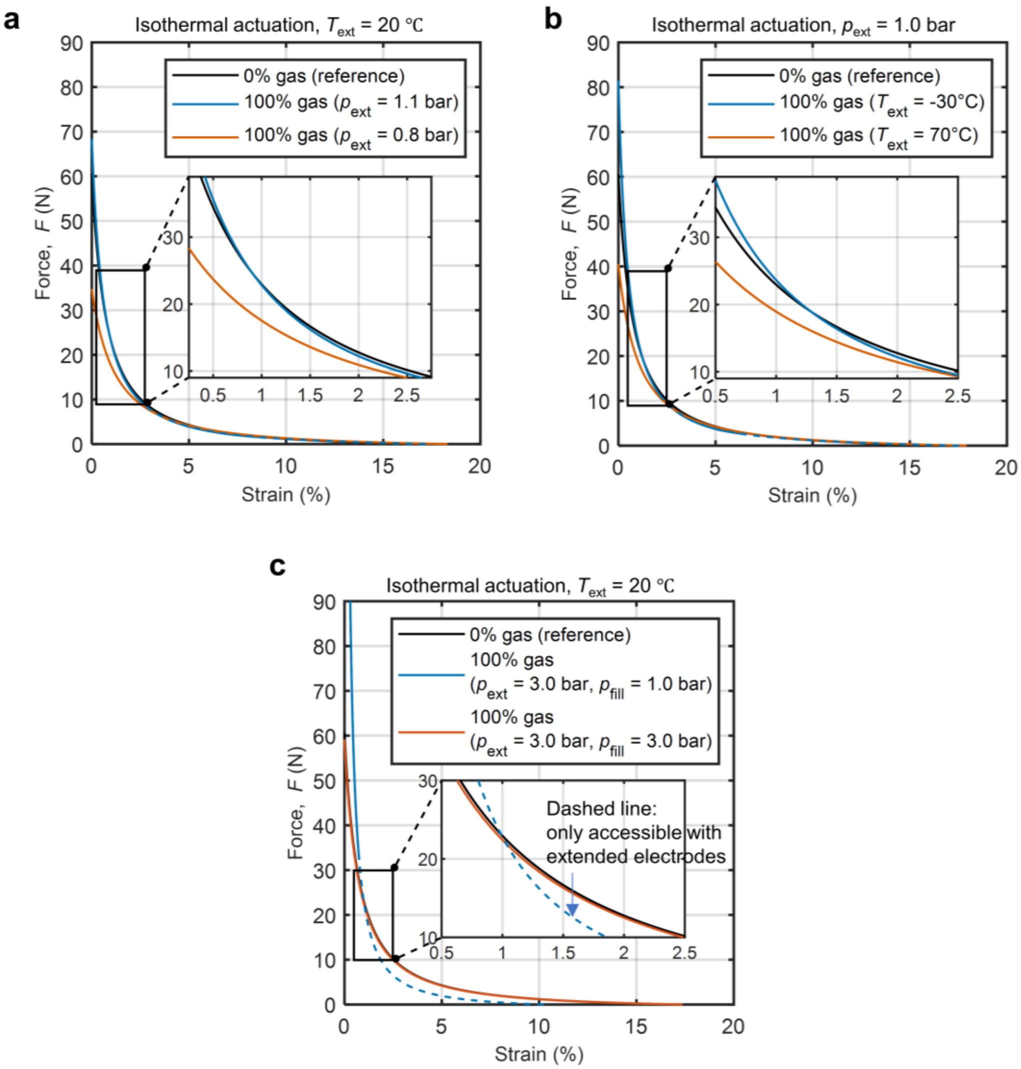

Supplementary Figure 18. Effects of external pressure and temperature during isothermal actuation.

a Calculated force-strain curves of a 100% gas-filled actuator under different external pressures (0.8 and 1.1 bar) at 20 °C, when the actuator is filled at a pressure p_0 = 1.0 bar and temperature T_0 = 20 °C. **b** Calculated force-strain curves under different external temperatures (-30 and 70 °C) at 1.0 bar, when the actuator is filled at a pressure p_0 = 1.0 bar and temperature T_0 = 20 °C. **c** Calculated force-strain curves under 3.0 bar external pressure at 20 °C, representing an underwater condition. When the actuator is initially filled at 1.0 bar, it exhibits reduced strain at low forces and enhanced strain at high forces due to the compressed internal gas volume. Under such high pressure, the gas volume considerably decreases, requiring extended electrodes to reach the cylindrical shape at fully zipped state, especially at low forces. When the actuator is filled at 3.0 bar to match

the external pressure, compressibility becomes negligible and the original force-strain behavior is recovered. Source data are provided as a Source Data file.

Supplementary Table 1. Coefficient for the additional mass under different external loads.

M (g)	α_0 (rad)	α_f (rad)	$\bar{c_1}$
10	0.24	1.5210	75.1368
20	0.24	1.4757	77.853
50	0.24	1.3616	85.6449
100	0.24	1.2197	97.7956
200	0.24	1.0334	120.0789
500	0.24	0.7614	178.9948

Supplementary References

1 Kellaris, N., Venkata, V. G., Rothemund, P. & Keplinger, C. An analytical model for the design of Peano-HASEL actuators with drastically improved performance. *Extreme Mech. Lett.* **29**, 100449, doi:10.1016/j.eml.2019.100449 (2019).

2 Rothemund, P., Kellaris, N. & Keplinger, C. How inhomogeneous zipping increases the force output of Peano-HASEL actuators. *Extreme Mech. Lett.* **31**, 100542, doi:10.1016/j.eml.2019.100542 (2019).

3 Kellaris, N., Gopaluni Venkata, V., Smith, G. M., Mitchell, S. K. & Keplinger, C. Peano-HASEL actuators: Muscle-mimetic, electrohydraulic transducers that linearly contract on activation. *Sci. Robot.* **3**, eaar3276, doi:10.1126/scirobotics.aar3276 (2018).

4 Paschen, F. *Ueber die zum funkenübergang in luft: wasserstoff und kohlensäure bei verschiedenen drucken erforderliche potentialdifferenz.* (JA Barth, 1889).

5 Rothemund, P., Kirkman, S. & Keplinger, C. Dynamics of electrohydraulic soft actuators. *Proc. Natl. Acad. Sci. USA* **117**, 16207-16213, doi:10.1073/pnas.2006596117 (2020).

6 Sîrbu, I.-D. *et al.* Electrostatic actuators with constant force at low power loss using matched dielectrics. *Nat. Electron.* **6**, 888-899 (2023).
