# Supplementary material for: Ultralight soft electrostatic actuators based on solid-liquid-gas architectures
Source: Nat Commun. 2026 Feb 19;17:1929. doi: 10.1038/s41467-026-69463-4 (PMC12923549; doi:10.1038/s41467-026-69463-4)
Supplement: Supplementary file 2 — Description of Additional Supplementary Files [file 41467_2026_69463_MOESM2_ESM.pdf]

File Name: Supplementary Movie 1

Description: Ultralight soft electrostatic actuators based on solid-liquid-gas architectures. The video is played in real time.

File Name: Supplementary Movie 2

Description: Soft electrostatic actuators based on solid-liquid-gas with different air-fills at different actuation frequencies. The video is played at 0.0125 speed.

File Name: Supplementary Movie 3

Description: Actuators using solid-liquid-gas architectures enable robotic systems with higher agility. The video is played at 0.05 speed.
